# Gynecology

**Published:** 1876-08

**Authors:** 


### I. GYNECOLOGY.

1. *Harmlessness of grave Operations in Pregnancy.* NICAISE. (*Before the Paris Surgical Society*, March 8,)

A woman had periosteal sarcoma of the humerus so far developed that disarticulation was determined upon in spite of the fact that she was far advanced in pregnancy. No accident occured and labor supervened normally in five weeks. It was concluded that severe surgical operations in pregnancy are less dangerous than is commonly supposed.

Verneuil considered that the gravity was proportioned to the intensity of the traumatic fever. If the temperature rises above 40° C., abortion and all its complications may be feared. But if the traumatic fever is moderate, the prognosis in pregnancy is no less favorable than when there is no gestation.

Polaillon believed that two other factors were not without their influence. Abundant hemorrhage predisposed to grave accidents; and the period of gestation is to be considered, for the first four or five months are more to be feared in this respect than the latter half of the term of pregnancy.

Guéniot agreed with Verneuil, but thought that absolute conclusions could not be drawn from these statements. He had seen women arrive at term after passing through all the stages of pericranial erysipelas of the worst form. An element of uncertainty exists in the variable contractibility (physiological irritability?) of the uterus in different women. This difference explains why traumatism is not always followed by the same results in pregnancy.

2. *Cysts of the Vagina.* DUPUY. (*Gazette Obstet*, April 20.)

All authors agree as to the rarity of vaginal cysts. Judging, however, from the number of recently reported cases, it would seem that this rarity has been exaggerated. The first description of this disease is to be credited either to Sir Astley Cooper or to Okley Heming (1831). Hausmann, however, in Haller's *Elementa Physiologiæ Corporis Humani*, has discovered the following passage; "*Glandulas vaginæ veras, rotundas, varii viri reper-*

*erunt; etiam depinxerunt, in brutis frequentioras; tamen etiam in homine visas. Eas non reperi, estas hydatides* (a synonym for cyst in that day) *in vagina vidi, et rejecit Ill. Morgagni.*"

They are most often found in the anterior vaginal wall, near the urethra; they are, however, to be discovered elsewhere. A cyst was found in 1872 (service of Demarquay), in the posterior wall, and quite voluminous. There is no recorded observation of such in the lateral walls.

Hegar and Kaltenbach have determined the exact position of these tumors. They are either submucous, interstitial (between the laminæ of the muscular layer) or subserous and perivaginal [between the vagina and rectum, above Douglas' cul-de-sac]. Huguier believes they originate in obliterated follicles of the vaginal membrane, but Preuscheu declares they arise in two distinct forms: (*a*) simple invaginations; (*b*) the more numerous are deep and broad depressions, presenting narrow prolongations in the shape of the fingers of a glove.

The epithelial lining of the vagina is prolonged into the glands: in the excretory canal it is stratified; elsewhere in the glands it is ciliated and cylindrical. Vaginal cysts may be due to retention of secretion in these glands. When they are found in the excretory canal, the enveloping membrane is furnished interiorly with pavement epithelium; when, on the contrary, they are found in the gland proper, the epithelium is cylindrical.

A vaginal cyst may originate in a hæmatoma. Barnes describes hæmatoma of the vaginal parietes resulting in several cases from labor. The resorption of the blood leaves a pouch which later becomes filled with serum, or sero-pus.

Kolaczek saw a cyst formed by the dilatation of a lacuna of Morgagni, the orifice of communication with the canal of the urethra having been obliterated. The woman was twenty-six years old and had suffered from uterine prolapse for six years. A fluctuating tumor in front of the anterior lip projected beyond the vulva, interfering with locomotion, and yielded, on puncture, a thin mucous fluid, which continued to exude through a fistulous orifice. The cyst, after extirpation, displayed a very dense stratified pavement epithelium. Hence the diagnosis. The dimensions of these cysts vary from those of a pea to those of the fist, and they are sometimes larger. The enveloping membrane may be dense and of determinate borders; or it may be thin, without line of demarcation separating it from contiguous parts. The contents are most often clear colored (like synovial fluid); when ruptured or incised it may become fetid and occasion septicemic accidents.

Certain varieties contain gas (Winkel, Braun, Schröder, Breisky). Schröder's case was that of a pregnant woman, almost the entire portion of whose vagina was covered with confluent elevations, some bright red, others gray and transparent. When punctured small crepitation was heard, and no liquid escaped. Pavement epithelium of variable dimensions was found on their interior, in the midst of which were large rounded cells containing a finely granular protoplasm and beautiful vesicular nuclei.

Breisky's case suffered from vaginal blennorrhagia. The anterior half of the vagina was normal, but the remainder was a conglomerate of small tumors (half pea to small nut in size), rosy or pure white in color. Some had a central depression, the umbilicus and contour of bright red hue. The white, on puncture, gave exit to gas, the red only a few drops of clear serum.

Vaginal cysts interfere with menstruation and coitus, may cause dysmenorrhæa; when extragenital may be painful or interfere with motion.

Simple puncture and ordinary incision do not promise much in the way of treatment. Puncture and irritating injections, or incision and cauterization may be followed by intense reaction, paravaginal inflammation, parametritis, violent inguinal or iliac pain.

Excision of a part of the wall of the cyst may be followed by secondary hemorrhage, requiring the tampon and styptics.

The radical and certain method is total extirpation. A longitudinal incision is made in the long diameter of the tumor, and the vaginal and cystic walls separated by dissection. When the latter is dense, decortication without opening may be attempted. When the wall is thin this is almost impossible, and it is necessary to remove the cyst in its entirety not without considerable difficulty.

Extirpation is best attempted in small cysts with a resisting envelope. For the larger cysts of thin envelopes, extensive incision with cauterization or applications of the tincture of iodine are best employed.

---

### II. Therapeutics.

1. *The Electrolytic Treatment of Tumors.* Julius Althans. (London.) *Berl. Kl. Wochenschr.*, Allg. Med. Central. Zt., 35, 1876.

In consideration of the great difference in opinions about the therapeutic usefulness of electrolysis, the author gives a brief account of his latest observations in this field.

1. *Nævus.* It is most frequently found in the face and on the scalp, and electrolysis is the most appropriate means for its removal. It is superior to excision, because there is no hemorrhage; to injections of sesquichloride of iron, because it does not endanger the life; to the cauterization by nitric acid, because it can be localized better; to the subcutaneous ligation, because after the operation the child has no pain and the surgeon has no further trouble; over the galvanocaustic, because it does not leave any scar. The ordinary round, small and thin nævus usually yields to one single application, while the larger "portroine stains" require a number of sittings.

The current acts through needles, which set in rows, and connected with the poles of ten to fifteen Daniell's elements are thrust into the tissue of the naevus. As soon as the chain is closed the destruction of the tex-

